# HBHA-IGRA and cytotoxic mediators release assays for the diagnosis of cervical tuberculous lymphadenitis

**DOI:** 10.1128/spectrum.01638-23

**Published:** 2023-11-01

**Authors:** Soumaya Bchiri, Asma Bouzekri, Rym Ouni, Rim Lahiani, Emna Romdhane, Neira Dekhil, Sonia Ben Hamouda, Helmi Mardassi, Asma Ferjani, Emanuelle Petit, Véronique Corbière, Soumaya Rammeh, Françoise Mascart, Camille Locht, Mamia Ben Salah, Mohamed Ridha Barbouche, Chaouki Benabdessalem

**Affiliations:** 1 Laboratory of Transmission, Control and Immunobiology of Infections, Pasteur Institute of Tunis, Tunis, Tunisia; 2 Department of biological sciences, Faculty of Sciences of Tunis, Tunis, Tunisia; 3 Institut Pasteur de Tunis, University of Tunis El Manar, Tunis, Tunisia; 4 ENT Department, Charles Nicolle Hospital, Tunis, Tunisia; 5 Faculty of Medicine of Tunis, University of Tunis El Manar, Tunis, Tunisia; 6 Department of Pathology, Charles Nicolle Hospital, Tunis, Tunisia; 7 Laboratory of Molecular Microbiology, Vaccinology and Biotechnology Development, Pasteur Institute of Tunis, Tunis, Tunisia; 8 Laboratoire de Recherche Résistance Aux Antibiotiques, Faculté de Médecine de Tunis, Hôpital Charles Nicolle, Tunis, Tunisia; 9 U-1019—CIIL-Center of Infection and Immunity of Lille, Univ Lille, CNRS, Inserm, Université de Lille, Institut Pasteur de Lille, Lille, France; 10 Laboratory of Vaccinology and Mucosal Immunity, Internal Medicine Department, Hôpital Universitaire de Bruxelles–CUB Hôpital Erasme, Université Libre de Bruxelles (U.L.B.), Brussels, Belgium; 11 Department of Microbiology, Immunology, and Infectious Diseases, College of Medicine and Medical Sciences, Arabian Gulf University, Manama, Bahrain; Quest Diagnostics Nichols Institute, Chantilly, Virginia, USA

**Keywords:** tuberculosis, cervical lymphadenitis, diagnosis, biomarkers, HBHA

## Abstract

Cervical tuberculous lymphadenitis (CTL), the most frequent extrapulmonary form of tuberculosis, is a serious health problem in Tunisia. CTL diagnosis is challenging mainly due to the paucibacillary nature of the disease and the potential misdiagnosis as cervical non-tuberculous lymphadenitis (CNTL). Here, we evaluated the performance of heparin-binding hemagglutinin (HBHA) interferon-gamma (IFN-γ) release assay (IGRA) for the diagnosis of CTL. In addition, we evaluated granzyme B, granulysin, and perforin release assays as CTL biomarkers and assessed their potential contribution to improve HBHA-IGRA performance. Peripheral blood mononuclear cells from CTL-suspected patients were stimulated with HBHA, early secreted antigenic target 6 (ESAT-6), or purified protein derivative (PPD) for 24 h in the presence of IL-7. Cytotoxic mediators and IFN-γ release were assessed by enzyme-linked immunosorbent assay. Receiver operating characteristic curves were used to evaluate the capacity of HBHA, ESAT-6, and PPD to discriminate between CTL (*n* = 27) and CNTL (*n* = 21). After applying bivariate and multivariate analyses, IFN-γ responses to HBHA appeared to offer the best distinction between CTL and CNTL, with an area under the curve of 0.9947, associated with 95.24% and 100% sensitivity and specificity, respectively. A principal component analysis showed clear clustering of the CTL versus the CNTL groups. This clustering was mainly attributed to HBHA-induced IFN-γ, PPD-induced granzyme B, and PPD-induced IFN-γ. These results thus suggest that the HBHA-IGRA provides high diagnostic accuracy for CTL versus CNTL, with high sensitivity and specificity. Combining HBHA-induced IFN-γ and PPD-induced granzyme B improves the accuracy to identify CTL.

**IMPORTANCE:**

Cervical tuberculous lymphadenitis (CTL), the most frequent extrapulmonary form of tuberculosis, is currently a major health problem in Tunisia and in several regions around the world. CTL diagnosis is challenging mainly due to the paucibacillary nature of the disease and the potential misdiagnosis as cervical non-tuberculous lymphadenitis. This study demonstrates the added value of the heparin-binding hemagglutinin-interferon-gamma release assay as an immunoassay in the context of CTL.

## INTRODUCTION

Tuberculosis (TB) has been a scourge of humanity for thousands of years. Every year, 10 million people across the world are estimated to develop the disease, particularly in low-income countries. After the decline of the coronavirus disease 2019, TB is once again the leading cause of death from a single infectious agent. An estimated 10.6 million new cases of TB were reported in 2021, compared to 10.1 million in 2020, and 1.6 million people died of TB in 2021, according to the global tuberculosis report (1).

In Tunisia, there were 4,500 notified cases of TB in 2021, with an incidence rate of 36 per 100,000 people (2). According to the statistics from the Tunisian Ministry of Health, extrapulmonary TB (EPTB) cases are in a constant increase, from 44.6% in 2011 to 62% of all TB cases to date. The most common form of EPTB in Tunisia is cervical tuberculous lymphadenitis (CTL), with an estimated 75% of the notified EPTB cases (3), and 76% of CTL cases are due to infection with *Mycobacterium bovis*. The diagnosis of CTL is challenging (4) because cervical lymphadenitis may occur in TB, as well as in other diseases, including cancer, other infectious etiologies, sarcoidosis, and other inflammatory conditions, which share many clinical manifestations with CTL (5). Therefore, imaging and evaluation of clinical symptoms are insufficient to conclusively diagnose CTL. Furthermore, the paucibacillary nature of CTL results in low sensitivity of bacteriological diagnostic tests (6, 7). As a result, invasive sampling, such as fine needle aspiration and biopsies, is often required for a decisive diagnosis (8). Hence, there is an urgent need for alternative reliable and rapid assays with high accuracy to discriminate CTL from cervical non-tuberculous lymphadenitis (CNTL).

Interferon-gamma (IFN-γ) release assay (IGRA), an *in vitro* immuno-diagnostic test, is based on the detection of IFN-γ responses to *Mycobacterium tuberculosis (Mtb*) complex antigens (Ag)s encoded in the region of difference 1, in particular, 10-kDa culture filtrate protein (CFP-10) and 6-kDa early secreted antigenic target 6 (ESAT-6) (9, 10). Many studies reported that IGRA is promising for the detection of TB infection but has a low ability to predict active TB (ATB) development (10). However, several studies revealed that IGRAs have limited performance in detecting an EPTB infection (11, 12). Nonetheless, other studies have examined the diagnostic accuracy of IGRA for EPTB, at various sites of infection, pointing out that the IGRA is a helpful tool for the diagnosis of tuberculous lymphadenitis (6, 13).

In order to enhance IGRA performances, other *Mtb* Ags were used as an alternative, such as heparin-binding hemagglutinin (HBHA) (14 – 16). HBHA is a methylated protein expressed by the members of the *Mtb* complex (17), and is involved in extrapulmonary dissemination (18), suggesting its potential role in EPTB diagnosis.

Many studies have demonstrated the potential value of HBHA-IGRA as a biomarker to distinguish TB infection from pulmonary TB (PTB) (19 – 21). However, these studies have mainly focused on PTB. Only a limited amount of data is available on EPTB (22, 23). Many studies focused on the utility of the use of tests based on HBHA for TB infection in subjects from different countries and at different ages including children, and in peripheral blood and at the lung site (24 – 27). To the best of our knowledge, no study has yet assessed the utility of HBHA-IGRA for the diagnosis of CTL, except in the recent study by Mascart et al. (28).

Here, we evaluated the diagnostic performances of HBHA-IGRA, as well as specific cytotoxic mediators release assays, by measuring *in vitro* the release of these effector molecules by peripheral blood mononuclear cells (PBMCs) after stimulation with purified protein derivative (PPD), ESAT-6 or HBHA from CTL and CNTL suspected Tunisian patients.

## MATERIALS AND METHODS

### Study population

Between 2018 and 2022, we enrolled, at Charles Nicolle Hospital in Tunis, 100 patients with CTL suspicion. Based on bacteriological, histological, and molecular findings, we did include 48 patients in the current study. The study was approved by the Pasteur Institute of Tunis Ethics Committee (2016/13C/I/CIC/V4). Written informed consent was obtained from all patients. Excision biopsy was performed to collect lymph nodes from CTL and CNTL patients. After an extensive diagnosis based on clinical symptoms, echography, histological, and bacteriological (29) arguments, patients were categorized into two groups: (i) CTL patients (*n* = 27) with confirmed TB based on the presence of epithelioid granulomas and/or necrosis as well as positive culture and/or positive GeneXpert. (ii) CNTL patients (*n* = 21). CNTL is a common disease occurring in patients of all ages. It is characterized by an abnormal enlargement of lymph nodes (>1 cm) in the head and neck. The CNTL group included nine cases of non-Hodgkin lymphoma, four cases of Hodgkin lymphoma, two cases with reactive lymph nodes, and six cases with TB suspicion, based on histopathological findings, but tuberculous and non-tuberculous mycobacteria were bacteriologically excluded based on culture and GeneXpert. Pregnant women were excluded from this study, as well as subjects with HIV infection, diabetes, autoimmune diseases, or under immunosuppressive treatment.

### PBMC isolation

PBMCs were purified from blood samples by density centrifugation using Ficoll-Paque (Eurobio, Les Ulis, France), washed twice in Hank’s Balanced Salt Solution (LONZA, Basel, Switzerland), and then resuspended at a concentration of 2 × 10^6^ cells per mL in Roswell Park Memorial Institute (RPMI)-1640 (LONZA, Basel, Switzerland) with 10% fetal bovine serum, 2 mM glutamine, 1× minimum essential medium nonessential AA, 1 mM Na-pyruvate, 50 µM 2-betamercaptophenol, and 40 µg/mL gentamicin (Sigma, MO, USA).

### PBMC stimulation and cytokine analyses in culture supernatants by ELISA

Cells were suspended at a density of 1 × 10^6^ viable cells per mL in RPMI-1640 complete medium in the presence of 1 ng/mL IL-7 (30), (207-IL, R&D Systems, Minneapolis, USA) in 5 mL polypropylene tubes (BD Biosciences, Becton, USA). Cells in each tube were stimulated either with 10 µg/mL HBHA (Institut Pasteur de Lille, Lille, France), 10 µg/mL ESAT-6 (Lionex, Braunschweig, Germany), or 4 µg/mL PPD (Statens Serum Institut, Copenhagen, Denmark). High antigen concentrations were used to measure cytotoxic mediators release, in addition to IFN-γ (31). Cells were also stimulated with 6 µg/mL phytohemagglutinin (PHA) (Sigma, MO, USA) used as a positive control, and an Ag-free medium condition was used as a negative control. Cells were cultured for 24 h at 37°C and in 5% CO2. Culture supernatants were collected to measure the following proteins: IFN-γ (Mabtech, Naka Strand, Sweden), granzyme B (Mabtech, Naka Strand, Sweden), perforin (Mabtech, Naka Strand, Sweden), and granulysin (R&D Systems, Minneapolis, USA). Enzyme-linked immunosorbent assays (ELISAs) were performed according to the manufacturer’s recommendations. The values obtained in the negative control condition were subtracted from concentrations of each protein or of the positive PHA control. Ag-specific responses were not considered when IFN-γ, granzyme B, perforin, or granulysin concentrations were below 1,000 pg/mL in response to PHA.

### Statistical analysis

Statistical analysis was performed using GraphPad Prism v8 (GraphPad Software). A nonparametric Mann-Whitney *U* test was applied to calculate the statistical significance between the two different groups. A *P-*value (*P*) < 0.05 was considered significant. The area under the receiver operating characteristic (ROC) curves (AUC) was performed for each analyte. Specificity and sensitivity were determined to select the best AUC based on the likelihood ratio. Principal component analysis (PCA) was performed using R program 4.1.3 (32).

## RESULTS

### Patient characteristics

Patients’ demographic and clinical characteristics are summarized in Table 1. The age range and the median are similar for both groups. However, the female-to-male ratio was 2.3 for the CTL group versus 0.61 for the CNTL group. This was expected since CTL affects women more than men in Tunisia. All patients were BCG vaccinated. It is worth noting that, based on echography, 92.6% of CTL patients presented unilateral adenopathy and 7.4% of patients presented bilateral adenopathy. Among the CNTL patients, 35.7% presented unilateral adenopathy and 64.3% patients presented bilateral adenopathy. In our study group, the difference between unilateral and bilateral lymphadenopathy in CTL and CNTL patients is statistically significant (*P* = 0.0002, using Fisher’s test). However, in current clinical practice, the presentation of unilateral versus bilateral lymphadenopathy has no diagnostic consequences for TB in cervical lymphadenitis to the best of our knowledge.

**TABLE 1:** Demographic and clinical characteristics

	CTL	CNTL
No. of subjects	*n* = 27	*n* = 21
Age [median (range)] (years)	47(18–77)	51(21–71)
Male/female (no./no.)	8/19	13/8
Smoking	5	6
Alcoholism	1	1
HIV	0	0
GeneXpert		
Positive (trace detected)	5	0
Positive (detected very low)	8	0
Positive (detected low)	8	0
Positive (detected)	6	0
Negative (not detected)	0	21

### Performance of HBHA-IGRA for the diagnosis of CTL

**Fig 1:**
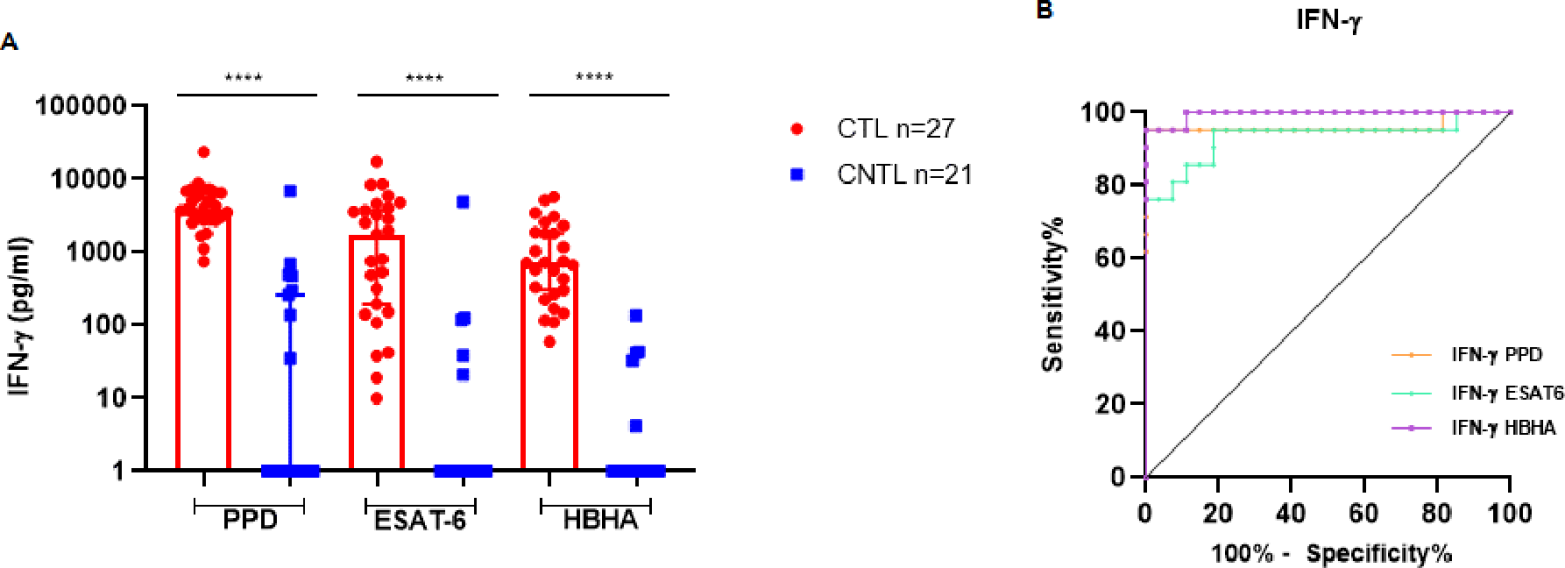
IFN-γ secretion in response to *Mtb* Ags in CTL versus CNTL patients. (**A**) Box plot presenting the median of *in vitro* IFN-γ secretion levels in response to *Mtb* Ags in CTL versus CNTL patients. Levels of IFN-γ were measured in the supernatant of PBMCs from CTL patients (*n* = 27) and CNTL patients (*n* = 21) after stimulation for 24 h in the presence of IL-7 with PPD/ESAT-6/HBHA. Statistical analysis was performed by the Mann-Whitney *U* test. (**B**) The ROC curve (plotting sensitivity versus 100% specificity) of IFN-γ to discriminate between CTL and CNTL patients is presented. ****P < 0.0001, ***P < 0.001, **P < 0.01, and *P < 0.05. pg/mL, picogram per milliliter and *N,* number of patients.

The HBHA-IGRA is a promising immunodiagnostic assay extensively used to distinguish between TB infection and PTB. Here, we assessed its diagnostic performance for CTL by measuring IFN-γ secretion by PBMCs from CTL and CNTL Tunisian patients after stimulation with *Mtb* Ags. As shown in Fig. 1A, PPD, ESAT-6, and HBHA induced high and comparable amounts of IFN-γ in the CTL group, which were significantly higher than in the CNTL group (*P* < 0.0001). However, the IFN-γ production in response to HBHA appeared to be the most distinctive between the CTL and CNTL groups, which was confirmed by the analysis of the AUC. We used ROC curves to comparatively evaluate the sensitivity and specificity of IFN-γ induced in response to PPD, ESAT-6, and HBHA to distinguish CTL from CNTL (Fig. 1B). ROC curves revealed that the IFN-γ production in response to HBHA allowed the best distinction of CTL from CNTL, as compared to PPD and ESAT-6, with an AUC of 0.9947 [95 % confidence interval (CI): 0.9819–1] (Table 2). The use of a cut-off value of 51.38 pg/mL gave the optimal combination between 95.24% of sensitivity (95% CI: 77.33%–99.76 %) and 100% of specificity (95% CI: 87.54%–100 %).

**TABLE 2:** Diagnostic performances of IFN-γ, granzyme B, granulysin, and perforin in response to PPD, ESAT-6, and HBHA

*Mtb* antigens	Biomarkers	AUC (95%CI)	*P*	Sensitivity (95%CI)	Specificity (95%CI)	Cut-off (pg/mL)
PPD	IFN-γ	0.9612 (0.8863–1.000)	<0.0001	95.24 (77.33–99.76)	100 (87.54–100)	718.6
	Granzyme B	0.9929 (0.9783–1.000)	<0.0001	95.24 (77.33–99.76)	96.30 (81,72-99,81)	844.1
	Perforin	0.7178 (0.5659–0.8697)	0.0103	71.43 (50.04–86.19)	74.07 (55.32–86.83)	173.7
	Granulysin	0.8660 (0.7609–0.9710)	<0.0001	61.90 (40.88–79.25)	88.89 (71.94–96.15)	286.4
ESAT-6	IFN-γ	0.9330 (0.8493–1.000)	<0.0001	80.95 (60–92.33)	92.59 (76.63–98.68)	29.59
	Granzyme B	0.9400 (0.8715–1.000)	<0.0001	80.95 (60–92.33)	92.59 (76.63–98.68)	55.51
	Perforin	0.7637 (0.6221–0.9053)	0.0019	71.43 (50.04–86.19)	81.48 (63.30–91.82	10.98
	Granulysin	0.8413 (0.7279–0.9547)	<0.0001	66.67 (45.37–82.81)	88.89 (71.94–96.15)	80.93
HBHA	IFN-γ	0.9947 (0.9819–1.000)	<0.0001	95.24 (77.33–99.76)	100 (87.54–100)	51.38
	Granzyme B	0.9409 (0.8689–1.000)	<0.0001	90.48 (71.09–98.31)	92.59 (76.63–98.68)	44.40
	Perforin	0.7981 (0.6683–0.9278)	0.0004	76.19 (54.91–89.37)	81.48 (63.30–91.82)	11.14
	Granulysin	0.7319 (0.5887–0.8752)	0.0063	61.90 (40.88–79.25)	74,07 (55.32–86.83)	31.73

### Performances of granzyme B, granulysin, and perforin release assays for the diagnosis of CTL

HBHA was shown to induce strong Th1 and cytotoxic CD8^+^ responses (33) as well as cytotoxic CD4^+^ responses (31), mainly in TB-infected subjects. Therefore, we evaluated the performances of cytotoxic mediators, namely granzyme B, granulysin, and perforin, in an attempt to further improve the HBHA-IGRA for the distinction between CTL and CNTL.

**Fig 2:**
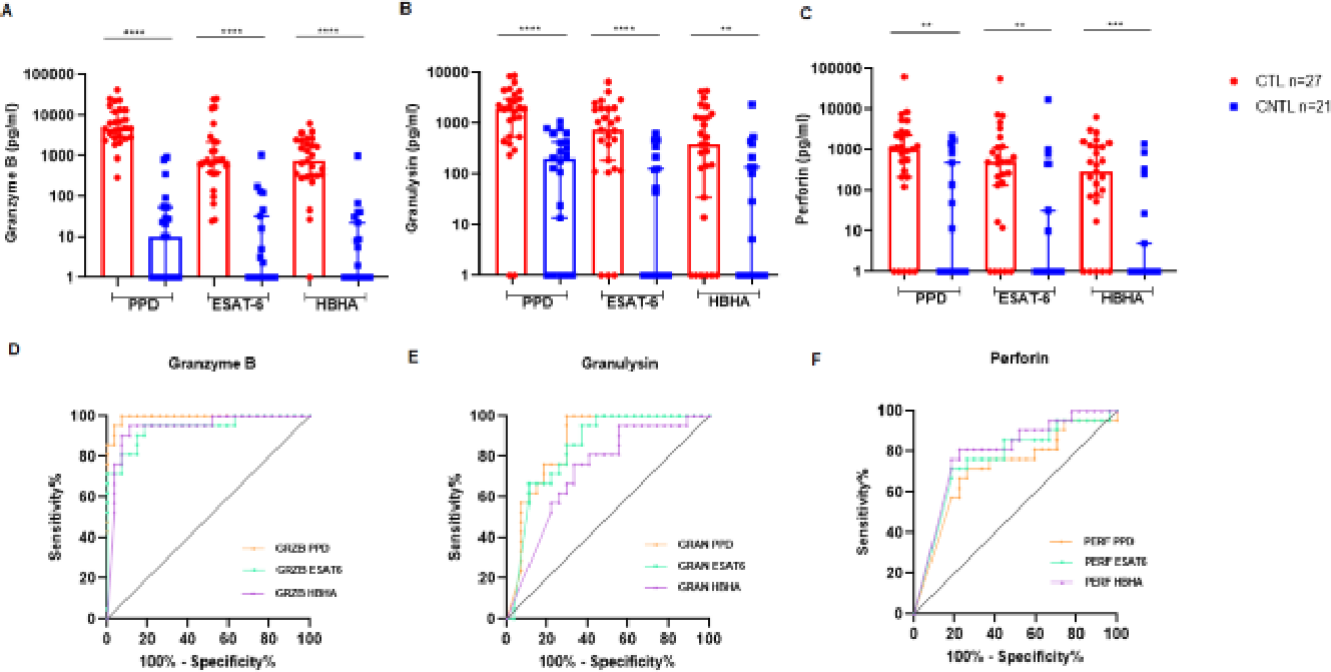
Cytotoxic mediators’ secretion in response to *Mtb* Ags in CTL versus CNTL patients. Box plot presenting the median of the *in vitro* release of granzyme B (**A**), granulysin (**B**), and perforin (**C**) in response to *Mtb* Ags in CTL versus CNTL patients. Levels of granzyme B, granulysin, and perforin were measured in the supernatant of PBMCs from CTL patients (*n* = 27) and CNTL patients (*n* = 21) after stimulation, for 24 h in the presence of IL-7, with PPD/ESAT-6/HBHA. Statistical analysis was performed by Mann-Whitney *U* test, *P* < 0.05. ROC curves (plotting sensitivity versus 100% specificity) of granzyme B (**D**), granulysin (**E**), and perforin (**F**) were performed. The cut-off value, AUC, sensitivity, specificity, and *P* are shown in Table 2. ****P < 0.0001, ***P < 0.001, **P < 0.01, and *P < 0.05. GRZB, granzyme B; GRAN, granulysin; PERF, perforin; pg/mL, picogram per milliliter; and *N*, number of patients.

Figure 2A through C shows that the levels of granzyme B, granulysin, and perforin following *Mtb* Ag stimulation were significantly higher in CTL patients than in CNTL patients. Overall, granzyme B, in response to PPD, ESAT-6, and HBHA, provided the best distinction, between CTL and CNTL, with a *P* < 0.0001. Granulysin, in response to PPD and ESAT-6, showed the highest distinction between CTL and CNTL (*P* < 0.0001), and somewhat lower for HBHA (*P* = 0.0046). In contrast, perforin, in response to HBHA, allowed the best distinction between CTL and CNTL (*P* = 0.0002) and less in response to PPD and ESAT-6, with a *P* of 0.0015 and 0.0011, respectively.

Among cytotoxic mediators, PPD-induced granzyme B exhibited the best distinction between CTL and CNTL (Fig. 2D) with an AUC of 0.9929 (95% CI: 0.9783–1). A cut-off value of 844.1 pg/mL gave the optimal combination between sensitivity (95.24%; 95% CI: 77.33%–99.76 %) and specificity (96%; 95% CI: 81.72%–99.81%) (Table 2). Figure 2E shows that PPD-induced granulysin provides a better distinction between CTL and CNTL with an AUC of 0.8660 (95% CI: 0.7609–0.9710) than in response to the other two antigens. The use of a cut-off value of 286.4 pg/mL gave 61.90% and 88.89% of sensitivity and specificity, respectively. For perforin (Fig. 2F), the best distinction between the two groups was found using HBHA stimulation (AUC = 0.78; 95% CI: 0.6683–0.9278). The use of a cut-off value of 11.14 pg/mL yielded a sensitivity of 76.17% (95% CI: 54.91%–89.37 %) and a specificity of 81.48% (95% CI: 63.30%–91.82 %), as shown in Table 2. Although there is an overlap in CI, the AUC of HBHA-IGRA was higher than those observed for the other biomarkers, with good sensitivity and specificity of 95.24% and 100%, respectively.

### Principal component analysis performed on *Mtb* Ag-induced markers

**Fig 3:**
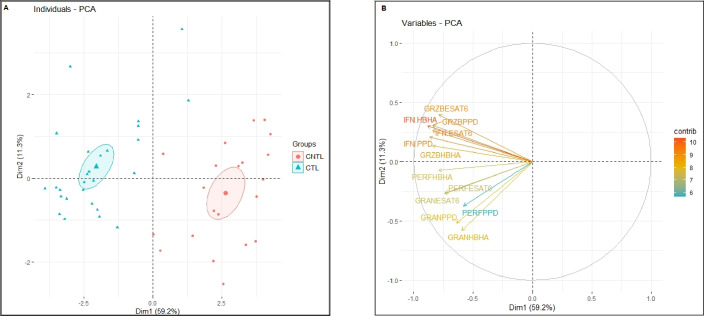
Principal component analysis performed on *Mtb* Ag-induced markers. (**A**) Variables show a distinct cluster between CTL and CNTL. The samples are represented by dots. Each color represents a group of patients. (**B**) The vectors represent the most influencing variables for each of the principal components PC1 (Individuals-PCA) and PC2 (Variables-PCA). The longer the arrow is, the more it influences the variance. The ellipses indicate 95% confidence. GRZB, granzyme B; GRAN, granulysin; and PERF, perforin.

We performed a PCA analysis to assess to which extent the studied biomarkers can contribute to the distinction between CTL and CNTL. Figure 3A shows good clustering of CTL versus CNTL groups using these biomarkers. This clustering was mainly attributed to HBHA-induced IFN-γ, PPD-induced granzyme B, and PPD-induced IFN-γ secretions (Fig. 3B). In addition, the individual plot showed a scattered distribution in the CNTL group, highlighting its heterogeneity in comparison to the assembled distribution of the CTL group.

### Diagnostic contribution of combined HBHA-induced IFN-γ and PPD-induced granzyme B for the distinction between CTL and CNTL

**Fig 4:**
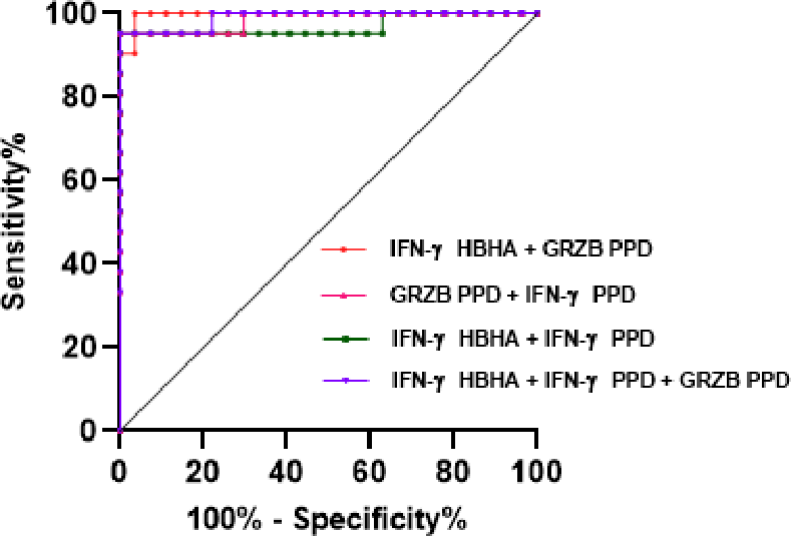
ROC curves’ analysis (plotting sensitivity versus 100% specificity) of combined AUC, to discriminate between CTL and CNTL. The cut-off value, AUC, sensitivity, specificity, and *P* are shown in Table 3. GRZB, granzyme B.

Based on the above PCA analysis, we have selected the top three ranked biomarkers that contribute to the distinction between CTL and CNTL. They are HBHA-induced IFN-γ, PPD-induced granzyme B, and PPD-induced IFN-γ. To investigate whether the combination of these biomarkers could improve the diagnosis of CTL, we calculated the AUC of different combinations (Table 3) and found that the AUC for HBHA-induced IFN-γ + PPD-induced granzyme B (Fig. 4) was 0.9965, with the highest combination of sensitivity (100%) and specificity (96.3%).

**TABLE 3:** Different combinations of AUC of Ag-specific biomarkers to improve the HBHA-IGRA

Combined biomarkers	AUC (95%CI)	*P*	Sensitivity (95%CI)	Specificity (95%CI)	Cut-off (pg /mL)
IFN-γ-HBHA + GRZB *^a^*-PPD	0.9965 (.9871–1.000)	<0.0001	100.0 (84.54–100)	96.30 (81.72–99.81)	487.1
IFN-γ-HBHA + IFN-γ-PPD	0.9700 (0.9114–1.000)	<0.0001	95.24 (77.33–99.76)	100 (87.54–100)	436.1
GRZB-PPD + IFN-γ-PPD	0.9859 (0.9566–1.000)	<0.0001	95.24 (77.33–99.76)	100 (87.54–100)	902.2
GRZB-PPD + IFN-γ-PPD + IFN-γ-HBHA	0.9894 (0.9666–1.000)	<0.0001	95.24 (77.33–99.76)	100 (87.54–100)	626.8

^*a*^ GRZB: granzyme B.

## DISCUSSION

CTL is a health threat in Tunisia, and despite advances in molecular diagnostic tools, especially for patients without a history of TB, the diagnosis of CTL remains challenging (7). In the differential diagnosis of CTL, other granulomatous lymphadenitis, such as those caused by other bacterial diseases such as sarcoidosis, toxoplasmosis, tularemia, fungal disease, cat-scratch disease, and neoplasms, must be taken into consideration (8). Therefore, the development of a fast, easy, reliable, and cost-effective diagnostic test will help in the early diagnosis of CTL, thereby allowing early initiation of treatment before a final diagnosis can be made through biopsy and culture (4).

An HBHA-IGRA is an already well-known promising immunodiagnostic test, mainly designed for the detection of TB infection status and its differential diagnosis from PTB (10, 14, 19, 21). HBHA is a surface-associated protein involved in adherence to epithelial cells, which has been shown to be required for extrapulmonary dissemination of *Mtb* (17, 18). Appropriate detection of PTB patients may, in contrast, only be achieved by combining ESAT-6-induced IP-10 with HBHA-induced IL-2 and GM-CSF in individuals already identified as *Mtb* infected by a combined HBHA and ESAT-6-IGRA (34). Alternatively, evaluation of the production of IFN-g induced by HBHA by lymphocytes collected at the site of infection may be a valuable aid to diagnose EPTB and PTB but is still poorly standardized (35). However, even though limited information is available, blood immune-based diagnosis may be different for EPTB compared to PTB. In contrast to PTB characterized most often by low HBHA-induced IFN-γ concentrations among PBMCs, HBHA may induce higher IFN-γ concentration secretion in patients with EPTB (28 and L. Aerts, personal communication). Thus, HBHA-blood-based tests may offer in some cases a valuable tool for diagnosis, particularly in cases where the infection is located in tissues outside the lungs, when traditional diagnostic strategies, such as sputum-based tests, may not be effective.

Our results indicate that HBHA-IGRA has identified the 27 CTL patients included in this study, whereas this IGRA was positive and with lower IFN-γ concentrations only in 4/21 CNTL patients. Similar numbers of positive results were found with the ESAT-6-IGRA, but the ESAT-6-induced IFN-γ concentrations were less discriminant than the HBHA-induced IFN-γ concentrations. The four CNTL patients with both a positive IFN-γ response to HBHA and to ESAT-6 were thus probably TB-infected patients, as also suggested by positive tuberculin skin testing for three of them. In contrast, the remaining 17 CNTL patients had negative HBHA and ESAT-6- IGRA, even though they were BCG vaccinated. Even considering that four CNTL patients present a TB infection, the HBHA-IGRA provided 95.4% sensitivity with 100% specificity for the diagnosis of cervical CTL. This largely exceeds the target product profile (TPP) for the TB diagnosis set by the WHO (sensitivity >80%, specificity >98% for a single test) (36). Results obtained with the HBHA-IGRA also displayed better performances than those obtained with commercial IGRAs that were analyzed in a meta-analysis from 10 published studies with pooled estimates of sensitivity and specificity of 89% and 81%, respectively, and an AUC of 0.93 (37). We show here the high potential diagnostic value for CTL in measuring the concentrations of PPD/ESAT-6/HBHA-induced IFN-γ with all the included patients being identified.

The HBHA-IGRA provided the best distinction between CTL and CNTL in this small cohort comprising only four probable TB-infected subjects among the CNTL, which is a limitation of this study. This diagnostic performance of the HBHA-IGRA to differentiate CTL from TB-infected subjects with CNTL should be confirmed in larger cohorts of patients including higher numbers of TB-infected subjects with different immune profiles. Whereas ESAT-6-induced IFN-γ concentrations may be higher than HBHA-induced IFN-γ concentrations in TB-infected subjects with actively multiplying bacteria, HBHA-induced IFN-γ concentrations are higher in TB-infected subjects who better control the infection (21, 38). In this study, among the three antigens, HBHA induced the lowest levels of IFN-γ in the CNTL group. Even if this suggests that HBHA may allow a better distinction between the studied groups than the other two Ags, these results may be due to the immune profile of the CNTL with TB infection. Our results are, however, in line with those recently reported in isolated CTL from patients living in a low TB-incidence country (28).

It has been shown that HBHA induces polycytotoxic CD4^+^ T lymphocytes to simultaneously produce IFN-γ along with granzymes, perforin and granulysin, both in TB-infected subjects and in patients with EPTB, but not in patients with PTB (31).

Few studies have explored the potential use of effector molecules produced by cytotoxic T lymphocytes for the immunodiagnosis of TB. Savolainen et al. have demonstrated that ESAT-6 induces high levels of IFN-γ and granzyme B, but there was no significant difference between PTB and TB-infected subjects (39). In contrast, we previously showed that Rv0140, a latency-associated Ag of *Mtb*, induces high granzyme B levels secreted mainly by CD8^+^ T cells derived from TB-infected individuals as compared to PTB patients. Consequently, we proposed the use of Rv0140-induced granzyme B as a discriminative biomarker of TB infection versus active disease (40). Similarly, high concentrations of granzyme A detected in plasmas from the commercialized QuantiFERON test were suggested to be biomarkers for TB infection (41). Other studies have focused on the use of *Mtb* Ag-induced IFN-γ and cytotoxic molecules as biomarkers to monitor the outcome of anti-TB treatment. Jiang et al*.* showed that after ESAT-6 and CFP-10 stimulation, the expression of perforin was significantly decreased and IFN-γ was significantly increased in patients with PTB compared to patients after 2 months of anti-TB treatment. Their results thus suggested that *Mtb* Ag-stimulated perforin downregulation and IFN-γ upregulation might be a potential index for monitoring therapy responses in ATB patients (42). In contrast, Pitabut et al*.* reported that IFN-γ, perforin, and granulysin levels were significantly increased after anti-TB treatment in TB or HIV/TB patients upon PPD or H37Ra stimulation of their PBMCs, suggesting that these mediators may serve as immune markers for the prediction of PTB, and as prognostic markers for therapeutic efficacy (43). Similarly, serum granulysin levels were reported as potentially useful to monitor treatment efficacy in childhood TB, being low before treatment and normalized at 4 months after completion of therapy (44). In the present study, we show that HBHA induces high levels of cytotoxic mediators, mainly, by PBMCs derived from CTL patients, and our data suggest that *Mtb* Ag-induced granzyme B is a promising discriminative biomarker between CTL and CNTL. ROC curve analyses highlighted that PPD-induced granzyme B displayed the highest AUC (0.9929). PPD-induced granulysin also discriminated better between the two groups (AUC = 0.8660) than granulysin induced by the other tested Ags. As for perforin, the highest AUC (0.7981) was obtained in response to HBHA.

Multivariate analyses showed a clear, distinct clustering of the two studied groups. Our results indicate the preponderant role of HBHA-induced IFN-γ, PPD-induced granzyme B, and PPD-induced IFN-γ as the major contributors to the segregation of CTL and CNTL. The scattered distribution within the CNTL group reflects the fact that this group included different cervical lymphadenitis etiologies other than TB. Moreover, when combining results from the HBHA-IGRA to those from the PPD-induced granzyme B release, we obtained 100% sensitivity with 96.3% specificity to identify cervical CTL.

The HBHA-IGRA test has certain limitations when it comes to TB-HIV co-infection. It has been shown that HIV infection significantly impairs the IFNγ expression in response to HBHA in the CD4^+^ T cells (26). On the other hand, Zou et al. showed that TB-HIV co-infections resulted in a marked increase of CD4lowCD8high subpopulation that have the same cytotoxic function as CD8^+^ T cells (45). The reason why we combined, in the current study, HBHA-IGRA with specific cytotoxic mediators’ release assays was in order to overcome this limitation.

In summary, we show here that HBHA-IGRA displayed a good performance to discriminate CTL from CNTL. Based on PCA analysis, we combined cytotoxic biomarkers to further improve the diagnostic accuracy of HBHA-IGRA. Our findings demonstrate that the assessed biomarkers exceeded those of the TPP for diagnostic TB detection set by the WHO, suggesting their usefulness for the differential diagnosis of CTL, once confirmed in a larger cohort.

## References

[1] Bagcchi S . 2023. WHO's global tuberculosis report 2022. Lancet Microbe 4:e20. doi:10.1016/S2666-5247(22)00359-7 36521512

[2] TB profile . 2023. Available from: https://worldhealthorg.shinyapps.io/tb_profiles/?_inputs_&entity_type=%22country%22&lan=%22EN%22&iso2=%22TN%22

[3] Siala M , Cassan C , Smaoui S , Kammoun S , Marouane C , Godreuil S , Hachicha S , Mhiri E , Slim L , Gamara D , Messadi-Akrout F , Bañuls A-L . 2019. A first insight into genetic diversity of Mycobacterium bovis isolated from extrapulmonary tuberculosis patients in south tunisia assessed by spoligotyping and MIRU VNTR. PLoS Negl Trop Dis 13:e0007707. doi:10.1371/journal.pntd.0007707 31532767 PMC6750577

[4] Rumende CM , Hadi EJ , Tanjung G , Saputri IN , Sasongko R . 2018. The benefit of interferon-gamma release assay for diagnosis of extrapulmonary tuberculosis. Acta Med Indones 50:138–143. 29950533

[5] Gautam H , Agrawal SK , Verma SK , Singh UB . 2018. Cervical tuberculous lymphadenitis: clinical profile and diagnostic modalities. Int J Mycobacteriol 7:212–216. doi:10.4103/ijmy.ijmy_99_18 30198498

[6] Kim YK , Uh Y , Lee NS , Cho MY , Eom M , Kim HY . 2011. Whole-blood interferon-gamma release assay for diagnosis of tuberculous lymphadenitis. Tohoku J Exp Med 224:189–193. doi:10.1620/tjem.224.189 21673481

[7] Shetty D , Vyas D . 2022. Combination method for the diagnosis of tuberculous lymphadenitis in high burden settings. Surg Exp Pathol 5:11. doi:10.1186/s42047-022-00111-z

[8] Deveci HS , Kule M , Kule ZA , Habesoglu TE . 2016. Diagnostic challenges in cervical tuberculous lymphadenitis: a review. North Clin Istanb 3:150–155. doi:10.14744/nci.2016.20982 28058405 PMC5206468

[9] Pai M , Denkinger CM , Kik SV , Rangaka MX , Zwerling A , Oxlade O , Metcalfe JZ , Cattamanchi A , Dowdy DW , Dheda K , Banaei N . 2014. Gamma interferon release assays for detection of Mycobacterium tuberculosis infection. Clin Microbiol Rev 27:3–20. doi:10.1128/CMR.00034-13 24396134 PMC3910908

[10] Goletti D , Delogu G , Matteelli A , Migliori GB . 2022. The role of IGRA in the diagnosis of tuberculosis infection, differentiating from active tuberculosis, and decision making for initiating treatment or preventive therapy of tuberculosis infection. Int J Infect Dis 124 Suppl 1:S12–S19. doi:10.1016/j.ijid.2022.02.047 35257904

[11] Fan L , Chen Z , Hao X-H , Hu Z-Y , Xiao H-P . 2012. Interferon-gamma release assays for the diagnosis of extrapulmonary tuberculosis: a systematic review and meta-analysis. FEMS Immunol Med Microbiol 65:456–466. doi:10.1111/j.1574-695X.2012.00972.x 22487051

[12] Zhou X-X , Liu Y-L , Zhai K , Shi H-Z , Tong Z-H . 2015. Body fluid interferon-γ release assay for diagnosis of extrapulmonary tuberculosis in adults: a systematic review and meta-analysis. Sci Rep 5:15284. doi:10.1038/srep15284 26503802 PMC4621514

[13] Song K-H , Jeon JH , Park WB , Kim S-H , Park KU , Kim NJ , Oh M , Kim HB , Choe KW . 2009. Usefulness of the whole-blood interferon-gamma release assay for diagnosis of extrapulmonary tuberculosis. Diagn Microbiol Infect Dis 63:182–187. doi:10.1016/j.diagmicrobio.2008.10.013 19070449

[14] Locht C , Hougardy J-M , Rouanet C , Place S , Mascart F . 2006. Heparin-binding Hemagglutinin, from an extrapulmonary dissemination factor to a powerful diagnostic and protective antigen against tuberculosis. Tuberculosis (Edinb) 86:303–309. doi:10.1016/j.tube.2006.01.016 16510310

[15] Tang J , Huang Y , Jiang S , Huang F , Ma T , Qi Y , Ma Y . 2020. Quantiferon-TB gold plus combined with HBHA-induced IFN-Γ release assay improves the accuracy of identifying tuberculosis disease status. Tuberculosis (Edinb) 124:101966. doi:10.1016/j.tube.2020.101966 32866942

[16] De Maio F , Squeglia F , Goletti D , Delogu G . 2019. The mycobacterial HBHA protein: a promising biomarker for tuberculosis. Curr Med Chem 26:2051–2060. doi:10.2174/0929867325666181029165805 30378481

[17] Menozzi FD , Rouse JH , Alavi M , Laude-Sharp M , Muller J , Bischoff R , Brennan MJ , Locht C . 1996. Identification of a heparin-binding hemagglutinin present in mycobacteria. J Exp Med 184:993–1001. doi:10.1084/jem.184.3.993 9064359 PMC2192777

[18] Pethe K , Alonso S , Biet F , Delogu G , Brennan MJ , Locht C , Menozzi FD . 2001. The heparin-binding haemagglutinin of M. tuberculosis is required for extrapulmonary dissemination. Nature 412:190–194. doi:10.1038/35084083 11449276

[19] Hougardy J-M , Schepers K , Place S , Drowart A , Lechevin V , Verscheure V , Debrie A-S , Doherty TM , Van Vooren J-P , Locht C , Mascart F , Hill P . 2007. Heparin-binding-hemagglutinin-induced IFN-γ release as a diagnostic tool for latent tuberculosis. PLoS One 2:e926. doi:10.1371/journal.pone.0000926 17912342 PMC1991599

[20] Bayaa R , Ndiaye MDB , Chedid C , Kokhreidze E , Tukvadze N , Banu S , Uddin MKM , Biswas S , Nasrin R , Ranaivomanana P , Raherinandrasana AH , Rakotonirina J , Rasolofo V , Delogu G , De Maio F , Goletti D , Endtz H , Ader F , Hamze M , Ismail MB , Pouzol S , Rakotosamimanana N , Hoffmann J , HINTT working group within the GABRIEL network . 2021. Multi-country evaluation of RISK6, a 6-gene blood transcriptomic signature, for tuberculosis diagnosis and treatment monitoring. Sci Rep 11:13646. doi:10.1038/s41598-021-93059-1 34211042 PMC8249600

[21] Dirix V , Dauby N , Hites M , Watelet E , Van Praet A , Godefroid A , Petit E , Singh M , Locht C , Mascart F , Corbière V . 2022. Optimal detection of latent Mycobacterium tuberculosis infection by combined heparin-binding hemagglutinin (HBHA) and early secreted antigenic target 6 (ESAT-6) whole-blood interferon gamma release assays. J Clin Microbiol 60:e0244321. doi:10.1128/jcm.02443-21 35430897 PMC9116186

[22] Sun Z , Nie L , Zhang X , Li Y , Li C . 2011. Mycobacterial heparin-binding haemagglutinin adhesion-induced interferon & antibody for detection of tuberculosis. Indian J Med Res 133:421–425. 21537096 PMC3103176

[23] Wen H-L , Li C-L , Li G , Lu Y-H , Li H-C , Li T , Zhao H-M , Wu K , Lowrie DB , Lv J-X , Lu S-H , Fan X-Y . 2017. Involvement of methylated HBHA expressed from Mycobacterium smegmatis in an IFN-γ release assay to aid discrimination between latent infection and active tuberculosis in BCG-vaccinated populations. Eur J Clin Microbiol Infect Dis 36:1415–1423. doi:10.1007/s10096-017-2948-1 28429162

[24] Chedid C , Kokhreidze E , Tukvadze N , Banu S , Uddin MKM , Biswas S , Russomando G , Acosta CCD , Arenas R , Ranaivomanana PP , Razafimahatratra C , Herindrainy P , Rakotonirina J , Raherinandrasana AH , Rakotosamimanana N , Hamze M , Ismail MB , Bayaa R , Berland J-L , De Maio F , Delogu G , Endtz H , Ader F , Goletti D , Hoffmann J . 2020. Relevance of quantiferon-TB gold plus and heparin-binding hemagglutinin interferon-γ release assays for monitoring of pulmonary tuberculosis clearance: a multicentered study. Front Immunol 11:616450. doi:10.3389/fimmu.2020.616450 33603746 PMC7885528

[25] Sali M , Buonsenso D , D’Alfonso P , De Maio F , Ceccarelli M , Battah B , Palucci I , Chiacchio T , Goletti D , Sanguinetti M , Valentini P , Delogu G . 2018. Combined use of quantiferon and HBHA-based IGRA supports tuberculosis diagnosis and therapy management in children. J Infect 77:526–533. doi:10.1016/j.jinf.2018.09.011 30267797

[26] Chiacchio T , Delogu G , Vanini V , Cuzzi G , De Maio F , Pinnetti C , Sampaolesi A , Antinori A , Goletti D . 2017. Immune characterization of the HBHA-specific response in Mycobacterium tuberculosis-infected patients with or without HIV infection. PLoS One 12:e0183846. doi:10.1371/journal.pone.0183846 28837654 PMC5570327

[27] Delogu G , Chiacchio T , Vanini V , Butera O , Cuzzi G , Bua A , Molicotti P , Zanetti S , Lauria FN , Grisetti S , Magnavita N , Fadda G , Girardi E , Goletti D , Tyagi A . 2011. Methylated HBHA produced in M. smegmatis discriminates between active and non-active tuberculosis disease among RD1-responders. PLoS One 6:e18315. doi:10.1371/journal.pone.0018315 21479248 PMC3066236

[28] Mascart F , Hites M , Watelet E , Verschelden G , Meuris C , Doyen J-L , Van Praet A , Godefroid A , Petit E , Singh M , Locht C , Corbière V . 2023. Analysis of a combined HBHA and ESAT-6-interferon-γ-release assay for the diagnosis of tuberculous lymphadenopathies. J Clin Med 12:2127. doi:10.3390/jcm12062127 36983128 PMC10052338

[29] Romdhane E , Arfaoui A , Benabdessalem C , Ksentini M , Ferjani A , Dekhil N , Lahiani R , Bchiri S , Mardassi H , Barbouche M-R , Boutiba Ben Boubake I , Ben Salah M , Rammeh S . 2020. Performance of genexpert ultra in the diagnosis of tuberculous cervical lymphadenitis in formalin fixed paraffin embedded tissues. Tuberculosis (Edinb) 125:102012. doi:10.1016/j.tube.2020.102012 33128936

[30] Wyndham-Thomas C , Corbière V , Dirix V , Smits K , Domont F , Libin M , Loyens M , Locht C , Mascart F . 2014. Key role of effector memory CD4^+^ T lymphocytes in a short-incubation heparin-binding hemagglutinin gamma interferon release assay for the detection of latent tuberculosis. Clin Vaccine Immunol 21:321–328. doi:10.1128/CVI.00651-13 24391135 PMC3957667

[31] Aerts L , Selis E , Corbière V , Smits K , Van Praet A , Dauby N , Petit E , Singh M , Locht C , Dirix V , Mascart F . 2019. HBHA-induced polycytotoxic CD4^+^ T lymphocytes are associated with the control of Mycobacterium tuberculosis infection in humans. J Immunol 202:421–427. doi:10.4049/jimmunol.1800840 30559320

[32] PCA in R using factominer: quick scripts and videos - articles - STHDA. 2017. Available from: http://www.sthda.com/english/articles/22-principal-component-methods-videos/65-pca-in-r-using-factominer-quick-scripts-and-videos

[33] Temmerman ST , Place S , Debrie A-S , Locht C , Mascart F . 2005. Effector functions of heparin-binding hemagglutinin-specific CD8^+^ T lymphocytes in latent human tuberculosis. J Infect Dis 192:226–232. doi:10.1086/430930 15962217

[34] Dirix V , Collart P , Van Praet A , Hites M , Dauby N , Allard S , Racapé J , Singh M , Locht C , Mascart F , Corbière V . 2022. Immuno-diagnosis of active tuberculosis by a combination of cytokines/chemokines induced by two stage-specific mycobacterial antigens: a pilot study in a low TB incidence country. Front Immunol 13:842604. doi:10.3389/fimmu.2022.842604 35359958 PMC8960450

[35] Henrard S , Corbière V , Schandené L , Ducarme M , Van Praet A , Petit E , Singh M , Locht C , Dirix V , Mascart F . 2019. Proportions of interferon-γ-producing ascites lymphocytes in response to mycobacterial antigens: a help for early diagnosis of peritoneal tuberculosis in a low TB incidence country. PLoS One 14:e0214333. doi:10.1371/journal.pone.0214333 30946755 PMC6448922

[36] World Health Organization . 2014. High priority target product profiles for new tuberculosis diagnostics: report of a consensus meeting. World Health Organization, Geneva, Switzerland.

[37] Liu Q , Li W , Chen Y , Du X , Wang C , Liang B , Tang Y , Feng Y , Tao C , He J-Q . 2017. Performance of interferon-γ release assay in the diagnosis of tuberculous lymphadenitis: a meta-analysis . PeerJ 5:e3136. doi:10.7717/peerj.3136 28413722 PMC5391793

[38] Corbière V , Pottier G , Bonkain F , Schepers K , Verscheure V , Lecher S , Doherty TM , Locht C , Mascart F . 2012. Risk stratification of latent tuberculosis defined by combined interferon gamma release assays. PLoS One 7:e43285. doi:10.1371/journal.pone.0043285 22912846 PMC3422279

[39] Savolainen LE , Koskivirta P , Kantele A , Valleala H , Pusa L , Tuompo R , Westerlund-Wikström B , Tuuminen T . 2013. Cytotoxic response persists in subjects treated for tuberculosis decades ago. BMC Infect Dis 13:573. doi:10.1186/1471-2334-13-573 24308801 PMC4029532

[40] Ouni R , Gharsalli H , Dirix V , Braiek A , Sendi N , Jarraya A , Douik El Gharbi L , Barbouche M-R , Benabdessalem C . 2019. Granzyme B induced by Rv0140 antigen discriminates latently infected from active tuberculosis individuals. J Leukoc Biol 105:297–306. doi:10.1002/JLB.MA0318-117R 30211958

[41] Guggino G , Orlando V , Cutrera S , La Manna MP , Di Liberto D , Vanini V , Petruccioli E , Dieli F , Goletti D , Caccamo N . 2015. Granzyme A as a potential biomarker of Mycobacterium tuberculosis infection and disease. Immunol Lett 166:87–91. doi:10.1016/j.imlet.2015.05.019 26051682

[42] Jiang H , Gong H , Zhang Q , Gu J , Liang L , Zhang J . 2017. Decreased expression of perforin in CD8^+^ T lymphocytes in patients with Mycobacterium tuberculosis infection and its potential value as a marker for efficacy of treatment. J Thorac Dis 9:1353–1360. doi:10.21037/jtd.2017.05.74 28616288 PMC5465152

[43] Pitabut N , Dhepakson P , Sakurada S , Keicho N , Khusmith S . 2020. Coordinated In vitro release of granulysin, perforin and IFN-γ in TB and HIV/TB co-infection associated with clinical outcomes before and after anti-TB treatment. Pathogens 9:655. doi:10.3390/pathogens9080655 32823923 PMC7459825

[44] Di Liberto D , Buccheri S , Caccamo N , Meraviglia S , Romano A , Di Carlo P , Titone L , Dieli F , Krensky AM , Salerno A . 2007. Decreased serum granulysin levels in childhood tuberculosis which reverse after therapy. Tuberculosis 87:322–328. doi:10.1016/j.tube.2007.01.003 17379576 PMC2692947

[45] Zou S , Tan Y , Xiang Y , Liu Y , Zhu Q , Wu S , Guo W , Luo M , Shen L , Liang K . 2022. The role of CD4^+^CD8^+^ T cells in HIV infection with tuberculosis. Front Public Health 10. doi:10.3389/fpubh.2022.895179 PMC9195591 35712309

